# Influence of Inflammation on Red Blood Cell Lifespan in Peritoneal Dialysis Patients

**DOI:** 10.3390/jcm13237104

**Published:** 2024-11-24

**Authors:** Huiping Zhao, Yuchao Zhao, Bei Wu, Li Bai, Lixia Lu, Li Zuo

**Affiliations:** Department of Nephrology, Peking University People’s Hospital, No.11 Xizhimen South Street, Xicheng District, Beijing 100044, China

**Keywords:** peritoneal dialysis, red blood cell lifespan, albumin, neutrophil-to-lymphocyte ratio, inflammation

## Abstract

**Background and hypothesis**: Renal anemia is linked to a decreased lifespan of red blood cells. The factors influencing red blood cell lifespan (RBCLS) in peritoneal dialysis patients, particularly the connection between inflammation and RBCLS, are still not well understood. This cross-sectional study was conducted to investigate these relationships. **Methods**: Patients who had been undergoing peritoneal dialysis for more than 3 months were selected for this study. The carbon monoxide exhalation method was used to detect the life span of red blood cells. The patients were then divided into two groups based on whether the RBCLS was shorter than 75 days. General information, anemia-related indicators, and other laboratory indicators along with their treatment were compared between the two groups. The different indicators were then included in a logistic regression analysis to identify independent risk factors that influence the shortening of RBCLS. **Results**: A cohort of 59 peritoneal dialysis patients was examined, revealing a median RBCLS of 88 days. Of these patients, 39 exhibited a normal RBCLS, while 20 had a shortened lifespan. A comparison of the two groups indicated that patients with a shorter RBCLS exhibited lower levels of PD vintage (29.5 vs. 56.0, *p* = 0.031), albumin (34.62 ± 3.01 vs. 37.41 ± 3.60, *p* = 0.004), cholesterol (4.02 ± 0.54 vs. 4.55 ± 1.21, *p* = 0.026), and low-density cholesterol (2.19 ± 0.43 vs. 2.75 ± 0.87, *p* = 0.002), and a higher neutrophil-to-lymphocyte ratio (NLR) (3.05 vs. 2.61, *p* = 0.026) compared to those with a normal RBCLS. The logistic regression results indicated that PD vintage (OR 0.976, CI 0.958–0.999), albumin (OR 0.668, CI 0.514–0.867), low-density lipoprotein cholesterol (OR 0.046, CI 0.002–0.953), and NLR (OR 1.792, CI 1.016–3.162) were identified as independent risk factors influencing the shortening of RBCLS. **Conclusion**: Hypoalbuminemia, low LDL, and high NLR in peritoneal dialysis patients are identified as independent risk factors associated with a shortened RBCLS. **Key learning points**: RBCLS is reduced in both peritoneal dialysis and hemodialysis patients. The neutrophil-to-lymphocyte ratio (NLR) in peripheral blood, a simple and readily available laboratory indicator, can reflect the inflammatory status. **This study adds**: Nutritional status (albumin and LDL) and inflammatory status (NLR) are significant factors that impact the lifespan of red blood cells in peritoneal dialysis patients. **Potential impact**: This study presents novel findings on the relationship between chronic inflammation and RBCLS in patients with chronic kidney disease, highlighting the need for additional research in this area.

## 1. Introduction

Anemia is a prevalent complication in individuals with end-stage renal disease (ESRD). The development of anemia in ESRD patients is influenced by various factors, such as low levels of endogenous erythropoietin (EPO), iron deficiency, chronic inflammation, inadequate nutrient intake, and shortened red blood cell lifespan (RBCLS), among others. Previous studies have confirmed that RBCLS is reduced in both chronic kidney disease (CKD) stage 3–5 [1], peritoneal dialysis (PD) [2], and hemodialysis (HD) [2,3].

In CKD, the normal physiological processes that maintain RBC lifespan are disrupted. Patients with ESRD often experience oxidative stress due to the accumulation of uremic toxins and the activation of inflammatory pathways. This oxidative stress can lead to damage to RBC membranes and internal components, further contributing to their premature destruction. However, the direct impact of inflammation on RBCLS has not been conclusively established. Consequently, we aimed to investigate the relationship between inflammation and RBCLS through a cross-sectional study involving a cohort of peritoneal dialysis patients.

C-reactive protein and interleukin-6 are widely utilized in clinical settings to assess the inflammatory state. However, in typical patients, these biomarkers are not routinely monitored in the absence of overt infection. Recent studies have indicated that the neutrophil-to-lymphocyte ratio (NLR) in peripheral blood, a simple and readily available laboratory indicator, can reflect the inflammatory status in various chronic illnesses [4] and is linked to a poor prognosis [5]. In this study, we utilized the NLR as an indicator of inflammation to investigate whether the reduced RBCLS in patients undergoing peritoneal dialysis is associated with inflammatory processes. Additionally, we examined the risk factors that influence the shortened RBCLS.

## 2. Materials and Methods

### 2.1. Patients Selection

We conducted a cross-sectional study. Patients who met the inclusion criteria for this study were individuals aged 18 years and older with chronic renal failure who had undergone peritoneal dialysis for more than 3 months and received regular monthly follow-ups at the Department of Nephrology, Peking University People’s Hospital between June and August 2021. Exclusion criteria included a history of smoking, malignant tumors, active infections, blood system-related diseases, chronic lung disease, abnormal liver function, active bleeding, or severe bleeding tendencies. Approval for this study was obtained from the Ethics Committee of Peking University People’s Hospital under the reference number 2021PHB376-001 (Approval Date: 18 June 2021), and all participants provided informed consent.

All subjects used conventional glucose-based, lactate-buffered PD solutions (Ultrabag; Baxter Healthcare, Guangzhou, China) containing Mg^2+^ 0.25 mmol/L, Ca^2+^ 1.25 mmol/L or 1.75 mmol/L, Na^+^ 132 mmol/L, and Cl^−^ 96 mmol/L, administered via continuous ambulatory PD (CAPD) or intermittent PD with a daily dialysate exchange dose of 6 L to 10 L.

### 2.2. Clinical and Laboratory Data Collection

Patient’s clinical data include general information such as age, gender, cause of ESRD, duration on dialysis, presence of diabetes, body mass index (BMI), blood pressure, and the presence of residual renal function (urine output). Additionally, peritoneal dialysis-related details are provided, such as the PD mode, total daily dialysis dose, and ultrafiltration volume. The patient’s medication status covers treatments for renal anemia (erythropoietin/roxadustat).

Laboratory tests included hemoglobin (HGB), neutrophil-to-lymphocyte ratio (NLR), platelet-to-lymphocyte ratio (PLR), monocyte-to-lymphocyte ratio (MLR), serum albumin (ALB), corrected serum calcium (Ca), serum phosphorus (P), intact parathyroid hormone (iPTH), uric acid (UA), triacylglycerol (TG), low-density lipoprotein cholesterol (LDL-C), high-density lipoprotein cholesterol (HDL-C), total cholesterol (T-Cho), urea, creatinine (Cr), and total urea clearance index (Kt/V).

Detection of red blood cell lifespan: Levitt’s CO breath test was conducted to determine the RBCLS. The concentration of carbon monoxide (CO) in exhaled alveolar gas primarily arises from endogenous metabolism and exogenous inhalation, with approximately 70% of endogenous CO generated from the catabolism of hemoglobin released following RBC destruction. By eliminating the interference from exogenous CO, the lifespan of RBCs can be estimated based on the total amount of CO released by hemoglobin in the body, divided by the amount of CO produced through daily hemoglobin degradation [6]. Subjects are required to fast and abstain from smoking for 24 h prior to the test. Collection should be performed in the morning before 12 noon. The carbon monoxide (CO) exhalation method using the RBS-01A red blood cell life tester (Shenzhen Xianya Biotechnology Co., Ltd., Shenzhen, China) was employed. The procedure involves ① Connecting the gas collection device by linking the alveolar gas collection bag and the luminal gas bag with the blowing nozzle through a three-way duct. ② Gas collection: the patient holds the gas collection device, takes a deep breath, holds it for 10 s, and then exhales forcefully into the alveolar gas collection bag to eliminate dead space gas in the respiratory tract and collect the exhaled breath; simultaneously, environmental air samples are collected. Two gas samples are sent for inspection and the CO concentration is measured using the machine. Instrument operation must strictly adhere to the provided instructions.

The normal range for RBC lifespan is ≥75 days.

### 2.3. Statistical Analysis

Statistical analysis was conducted using SPSS 25.0 software. Patients were divided into two groups based on whether RBCLS was less than 75 days. For measurement data following a normal distribution, results were presented as mean ± standard deviation and compared using an independent sample t-test. Non-normally distributed data were presented as median [M (P25, P75)] and compared using the Mann–Whitney U test. Count data were expressed as rates or percentages, with comparison between groups carried out using χ2 test or Fisher’s exact test. Logistic regression was then conducted using the shortening of RBCLS as the dependent variable and the differing indicators between the two groups as covariates, in order to identify independent risk factors influencing the shortening of RBCLS. A significance level of *p* < 0.05 was considered statistically significant.

## 3. Results

### 3.1. Demographic Data and Clinical Characteristics

A total of 59 PD patients were included in this study with a mean age of 58.7 ± 11.2 years and a median dialysis duration of 46.0 (21.0, 84.0) months, comprising 29 male patients. The primary cause of ESRD was chronic glomerulonephritis in 26 individuals, followed by diabetic nephropathy in 18 individuals, hypertensive nephropathy in 5 individuals, chronic tubulointerstitial disease in 5 cases, and other causes in 5 individuals. The distribution of PD models was as follows: Continuous ambulatory peritoneal dialysis (CAPD) in 52 individuals, daytime intermittent peritoneal dialysis (DIPD) in 3 individuals, and automatic peritoneal dialysis (APD) in 4 individuals. Erythropoiesis stimulating agents (ESA) was administered to 27 patients, roxadustat to 26 patients, and a combination of roxadustat and ESA to 1 patient. Refer to Table 1 for further details.

### 3.2. Comparison Between Two Groups of Patients Based on Their Red Blood Cell Lifespan (RBCLS)

This study included 39 cases in the normal RBCLS group (RBCLS ≥ 75 days), with a median RBCLS of 101 days, and 20 cases in the shortened RBCLS group (RBCLS < 75), with a median RBCLS of 58 days.

Analysis revealed that patients with a shorter red blood cell lifespan had lower levels of PD vintage (29.5 vs. 56.0, *p* = 0.031), albumin (34.62 ± 3.01 vs. 37.41 ± 3.60, *p* = 0.004), cholesterol (4.02 ± 0.54 vs. 4.55 ± 1.21, *p* = 0.026), and low-density lipoprotein cholesterol (2.19 ± 0.43 vs. 2.75 ± 0.87, *p* = 0.002) compared to those with a normal red blood cell lifespan. Additionally, the neutrophil-to-lymphocyte ratio (NLR) was significantly higher in the shortened red blood cell lifespan group compared to the normal group (3.05 vs. 2.61, *p* = 0.026). However, there were no statistically significant differences in age, gender, medicines, hemoglobin, ferritin, transferrin saturation (TAST), calcium, phosphate, or parathyroid hormone between the two groups of patients. Refer to Table 2 for further details.

### 3.3. Analysis Focused on Independent Risk Factors Associated with Shortened RBCLS

After conducting collinearity testing, we found no evidence of collinearity between total cholesterol and low-density lipoprotein cholesterol, as indicated by a Variance Inflation Factor (VIF) of 1.99 for both variables. Statistically significant indicators in the comparison between the two groups (dialysis age, albumin, total cholesterol, low-density lipoprotein cholesterol, NLR) and clinically significant indicators (diabetes) were included in the logistic regression analysis. The results indicated that PD vintage (OR 0.976, 95% CI 0.958–0.999), albumin (OR 0.668, 95% CI 0.514–0.867), low-density lipoprotein cholesterol (OR 0.046, 95% CI 0.002–0.953), and NLR (OR 1.792, 95% CI 1.016–3.162) were identified as independent risk factors influencing the shortening of RBCLS. Refer to Table 3 for further details.

## 4. Discussion

The pathogenesis of renal anemia is primarily associated with a relative deficiency of EPO in the body, iron deficiency, shortened RBCLS, and chronic inflammation. The current main treatment options include exogenous ESA supplementation, hypoxia-inducible factor-prolyl hydroxylase inhibitors (HIF-PHIs), which regulate ESA production, and iron supplementation. Despite the use of ESA at doses exceeding conventional levels, some patients still exhibit poor anemia treatment outcomes. Research indicates that this may be linked to the shortened lifespan of red blood cells (RBCs) [7]. Consequently, an increasing number of scholars are focusing on studying the lifespan of RBCs in CKD patients. Li compared red blood cell lifespan in non-smoking CKD patients, and revealed that, as CKD advances, the lifespan of RBCs decreases. RBCLS in CKD stages 1–5 were 122 ± 50 days, 112 ± 26 days, 90 ± 32 days, 88 ± 28 days, and 60 ± 24 days, respectively. RBCLS means for the stage 3, 4, and 5 groups were significantly shorter than those for the stage 1 and 2 groups [1]. Vos FE investigated the RBCLS in 14 HD patients, 5 PD patients, and 14 healthy controls. The results indicated that the RBCLS for HD patients, PD patients, and healthy controls were 58.1 days, 55.3 days, and 72.9 days, respectively. There was no significant difference between HD and PD patients; however, both groups exhibited significantly shorter lifespans compared to healthy controls [2].

Numerous studies have investigated the factors contributing to the reduced lifespan of RBCs in ESRD. Oxidative stress plays a significant role in determining the lifespan of RBCs. This limited lifespan is largely due to the unique structure of RBCs, which lack nuclei and mitochondria, making them particularly vulnerable to oxidative damage from reactive oxygen species (ROS) generated during normal metabolic processes and environmental factors [8,9]. Uremic toxins, particularly indoxyl sulfate (IS) and p-cresol (PC), have been shown to significantly impact the lifespan of RBCs, particularly in patients with CKD. These toxins are known to induce oxidative stress, apoptosis, and alterations in cellular proliferation, which collectively contribute to accelerated aging of endothelial cells and, by extension, affect RBC longevity [10,11]. Inflammation has a significant impact on the lifespan of RBCs, primarily through mechanisms involving oxidative stress and apoptosis. Under inflammatory conditions, RBCs are exposed to increased levels of oxidative stress, which can lead to cellular injury and trigger a process known as eryptosis, or suicidal death of the cells. This process is characterized by the activation of calcium-permeable channels in the RBC membrane, leading to an influx of calcium ions. This influx subsequently causes membrane scrambling, exposing phosphatidylserine on the cell surface, which is a signal for phagocytosis by macrophages and other immune cells [12]. Diseases associated with chronic inflammation have been shown to correlate with increased oxidative stress and a higher rate of eryptosis [13].

Laboratory studies suggest that inflammation contributes to a shortened lifespan of RBCs. However, previous clinical studies provide only limited indirect evidence to support this conclusion. Among 339 hemodialysis patients, it was observed that those with elevated levels of high-sensitivity C-reactive protein (hsCRP) and IL-6 required a higher dosage of EPO, indicating a relationship between inflammation and reduced EPO response [14]. But neither of the two studies that directly investigated the relationship between inflammation and RBCLS produced positive results. In a study by Ma J et al., the correlation between the lifespan of RBCs and inflammatory factors was investigated in 54 hemodialysis patients. The results showed no significant correlation between the lifespan of RBCs and white blood cell count, hsCRP, IL-6, IL-18, and IL-10 [15]. Similarly, Bomholt. T compared clinical indicators and RBCLS in diabetic ESRD patients undergoing hemodialysis and diabetic non-renal patients, finding no association between RBCLS and inflammatory markers [16].

However, our study is the first to demonstrate that a high NLR is an independent risk factor associated with a shortened lifespan of RBCs. This study provides the first direct evidence that an elevated inflammatory state results in a reduced lifespan of RBCs.

This study also discovered that low serum albumin levels and low LDL levels are independent risk factors for shortened red blood cell lifespan (RBCLS), which aligns with the findings of a previous study by Sato Y et al. in hemodialysis patients [7]. Both studies indicate that poor nutritional status may be linked to a shorter lifespan of red blood cells.

There are no existing studies that have explored the connection between dialysis vintage and RBCLS. The findings of this research indicate that a shorter duration of dialysis is associated with a shorter RBCLS. This analysis may have been influenced by selection bias in the patient population included, as individuals with chronic inflammation and poor nutritional status often face higher early mortality rates and struggle to survive for extended periods. Consequently, patients who have undergone dialysis for a longer duration tend to be in better overall health, experiencing milder inflammation and better nutrition. As a result, these patients are more likely to have a normal RBCLS.

The limitations of this study include its cross-sectional design, potential selection bias, and the inability to establish a causal link between exposure factors (low albumin and high NLR) and disease (shortened RBCLS). Future research will involve a prospective cohort study to investigate the relationship between nutrition, inflammation, and prognosis in peritoneal dialysis patients.

## Figures and Tables

**Table 1:** Baseline demographic and clinical characteristics of enrolled PD patients (*n* = 59).

Variable	All (n = 59)
Age (years)	58.7 ± 11.2
Gender, male (n, %)	29 (49.2%)
PD vintage (months)	46.0 (21.0, 84.0)
BMI (kg/m^2^)	22.90 ± 3.03
SBP (mmHg)	130.95 ± 18.72
DBP (mmHg)	78.17 ± 12.36
Residual renal Kt/V (/w)	0.06 (0.00, 0.38)
Peritoneal KT/V (/w)	1.60 ± 0.46
Total Kt/V (/w)	1.83 ± 0.40
Diabetes (n, %)	18 (30.5%)
Medications for treating anemia	
ESA	27
Roxadustat	26
ESA + Roxadustat	1
No ESA and Roxadustat	5
HGB(g/L)	115.9 ± 11.6
NLR	2.82 (2.12, 3.46)
PLR	134.23 (103.47, 164.06)
MLR	0.36 (0.30, 0.47)
Ferritin (ng/mL)	243.90 ± 160.82
ALB (U/L)	36.47 ± 3.64
Ca (mmol/L)	2.40 ± 0.20
P (mmol/L)	1.46 ± 0.34
iPTH (pg/mL)	233.8 (97.5, 430.0)
UA (umol/L)	356.25 ± 57.3
TG (mmol/L)	2.26 ± 1.88
T-Cho (mmol/L)	4.38 ± 1.06
LDL-C (mmol/L)	2.56 ± 0.79
HDL-C (mmol/L)	1.10 ± 0.36
urea (mmol/L)	19.92 ± 5.96
Scr (umol/L)	974.03 ± 229
Glucose	6.69 ± 2.65
RBCLS (days)	88 (62, 108)

BMI: body mass index; SBP: Systolic blood pressure; DBP: Diastolic blood pressure; Kt/V: urea clearance; EPO: erythropoietin; HGB: hemoglobin; NLR: ratio of neutrophils to lymphocytes; PLR: ratio of platelets to lymphocytes; MLR: ratio of monocytes to lymphocytes; Alb: albumin; cCa: corrected calcium; P: phosphate; iPTH: intact parathyroid hormone; UA: uric acid; TG: triglyceride; T-Chol: total cholesterol; LDL-C: low-density lipoprotein cholesterol; HDL-C: high-density lipoprotein cholesterol; Sc: serum creatinine; and RBCLS: red blood cell lifespan.

**Table 2:** Comparison between the normal RBCLS group and the shortened RBCLS group.

	Normal RBCLS Group n = 39	Shortened RBCLS Group n = 20	t/Z/x^2^	P
RBCLS	101 (88, 117)	58 (42, 65)	−6.247	0.000
Age (years)	58.46 ± 11.20	59.25 ± 11.30	−0.255	0.800
Gender (male), n (%)	18 (45.2)	11 (55.0)	0.414	0.520
Diabetes, n (%)	11 (28.2)	7 (35.0)	0.288	0.592
PD vintage (months)	56.0 (25.5, 107.5)	29.5 (12.0, 69.0)	−2.153	0.031
BMI (kg/m^2^)	23.04 ± 2.99	22.68 ± 317	0.420	0.677
EPO, n (%)	22 (56.4)	6 (30.0)	3.698	0.097
Roxadustat, n (%)	15 (38.5)	12 (60.0)	2.471	0.168
Iron dosage (mg)	300 (150, 300)	300 (0, 300)	−0.864	0.387
Residual renal Kt/V (/w)	0.06 (0.00, 0.40)	0.07 (0.00, 0.37)	−0.017	0.987
Peritoneal KT/V (/w)	1.57 ± 0.38	1.66 ± 0.58	−0.593	0.558
Total Kt/V (/w)	1.79 ± 0.35	1.90 ± 0.48	−0.951	0.336
Anuria, n (%)	17 (43.6)	9 (45.0)	0.011	0.918
HGB (g/L)	116.81 ± 9.53	113.9 ± 14.92	0.796	0.433
WBC (10^9^/L)	7.21 ± 2.09	7.41 ± 2.36	−0.321	0.750
PLT (10^9^/L)	222.71 ± 54.59	210.35 ± 71.2	0.680	0.501
Ferritin (ug/L)	184.40 (98.85, 377.8)	251.50 (154.50, 323.30)	−0.467	0.684
TSAT (%)	29.02 (19.68, 43.26)	29.28 (17.28, 39.01)	−0.435	0.664
ALB (g/L)	37.41 ± 3.60	34.62 ± 3.01	2.966	0.004
Urea (mmol/L)	20.59 ± 6.12	18.63 ± 5.55	1.200	0.235
Scr (umol/L)	1003.74 ± 209.34	916.10 ± 260.77	1.399	0.167
cCa (mmol/L)	2.43 ± 0.14	2.34 ± 0.29	1.502	0.139
P (mmol/L)	1.45 ± 0.31	1.47 ± 0.39	−0.159	0.874
iPTH (pg/mL)	220.90 (108.22, 417.55)	248.50 (93.31, 461.70)	−0.508	0.612
UA (umol/L)	350.76 ± 54.33	366.95 ± 62.83	−1.027	0.309
Glucose	6.91 ± 2.93	6.24 ± 1.99	1.006	0.319
T-Chol (mmol/L)	4.55 ± 1.21	4.02 ± 0.54	2.282	0.026
TG (mmol/L)	2.21 ± 1.06	2.36 ± 2.93	−0.215	0.832
HDL-C (mmol/L)	1.11 ± 0.39	1.06 ± 0.30	0.526	0.601
LDL-C (mmol/L)	2.75 ± 0.87	2.19 ± 0.43	3.307	0.002
NLR	2.61 (1.89, 3.23)	3.05 (2.78, 3.64)	−2.233	0.026
PLR	133.99 (103.70, 161.19)	134.48 (101.42, 170.34)	−0.512	0.608
MLR	0.35 (0.27, 0.44)	0.38 (0.33, 0.63)	−1.602	0.109

Kt/V: total urea clearance; HGB: hemoglobin; WBC: white blood cell; PLT: platelet; TSAT: transferrin saturation; ALB: albumin; cCa: corrected calcium; P: phosphate; iPTH: intact parathyroid hormone; UA: uric acid; TG: triglyceride; T-Chol: total cholesterol; LDL-C: low-density lipoprotein cholesterol; HDL-C: high-density lipoprotein cholesterol; Sc: serum creatinine; NLR: ratio of neutrophils to lymphocytes; PLR: ratio of platelets to lymphocytes; MLR: ratio of monocytes to lymphocytes; and RBCLS: red blood cell lifespan.

**Table 3:** Independent risk factors associated with shortened RBCLS.

	B	SE	Wals	P	OR	95%CI
Diabetes	−1.253	0.986	1.615	0.204	0.286	0.041–1.973
PD vintage	−0.025	0.012	4.289	0.038	0.976	0.958–0.999
NLR	0.583	0.290	4.053	0.044	1.792	1.016–3.162
Albumin	−0.404	0.133	9.187	0.002	0.668	0.514–0.867
total cholesterol	1.123	1.130	0.988	0.320	3.075	0.336–28.170
LDL-C	−3.083	1.548	3.964	0.046	0.046	0.002–0.953
constant	16.142	5.655	8.149	0.004	10,241,924	

NLR: ratio of neutrophils to lymphocytes; LDL-C: low-density lipoprotein cholesterol.

## Data Availability

Dataset available on request from the authors.
